# Stronger associations between a Brief Evaluation of Activities of Daily Living and Cognition (BEAN) and cognitive activity than physical activity in unimpaired older adults

**DOI:** 10.1002/alz70857_104223

**Published:** 2025-12-25

**Authors:** Sydney Y Schaefer, Michael H. Malek‐Ahmadi, Angela Kuramoto, Farah Shaikh, Carla Jooste, Hamzah Al‐Jubouriy, Kathy O'Connor, David W. Coon, Alireza Atri

**Affiliations:** ^1^ Arizona State University, Tempe, AZ, USA; ^2^ Banner Alzheimer's Institute, Phoenix, AZ, USA; ^3^ Arizona Alzheimer's Consortium, Phoenix, AZ, USA; ^4^ Banner Sun Health Research Institute, Sun City, AZ, USA; ^5^ Arizona State University, Phoenix, AZ, USA

## Abstract

**Background:**

Cognitive and physical activity can reduce dementia risk among older adults. We have developed and validated a Brief Evaluation of Activities of Daily Living and Cognition (BEAN) test that is performance‐based, where a spoon is used to acquire and transport objects. It is unclear, however, whether its relationship to cognition and daily functioning is mediated through cognitive or physical (i.e., cardiovascular/musculoskeletal) mechanisms. Based on prior work, we hypothesized the BEAN test would be related to the level of engagement in cognitive but not physical activities.

**Method:**

Data were collected in the Banner Sun Health Research Institute's Center for Healthy Aging's Longevity Study of community‐dwelling adults age 50+ with no cognitive impairment (based on self‐report and Clinical Dementia Rating=0) and are independent in personal/home affairs (based on self‐report). A total of 290 participants (mean±SD age = 79.3±8.5 years; mean±SD education = 15.9±1.3 years; 71.7% female) completed the BEAN test during at least one annual visit. Participants also completed the Montreal Cognitive Assessment (MoCA) (97%), the Rapid Assessment of Physical Activity (RAPA) (95%), and the Cognitively Stimulating Activities Questionnaire (CSA‐Q) (81%), depending on when they were enrolled. Only cross‐sectional data were analyzed in this study. BEAN scores reflect intraindividual variability across four consecutive trials of the task (where higher variability is worse).

**Result:**

Generalized linear mixed models with a gamma distribution confirmed that the BEAN test and MoCa were related (b=‐.68; *p* = .0025), controlling for age, sex, and education. The BEAN test was also related to the CSA‐Q (b=‐.24; *p* = .02). Furthermore, mediation analysis showed that the BEAN test had a negative total effect (b=‐0.07; *p* = .0002) on MoCA that was mediated through the CSA‐Q (b=‐.12; *p* = .0033). However, the BEAN test and RAPA were not related (*p* = .50), and RAPA scores did not mediate the relationship between BEAN scores and MoCA scores (*p* = .10).

**Conclusion:**

Results support the use of the BEAN test as a measure of cognition and daily functioning rather than as a physical/motor assessment. Furthermore, the BEAN test appears to be sensitive to the mechanisms by which cognitive engagement, rather than physical activity, positively impact cognition in community‐dwelling older adults.

